# A Temporal Neural Trace of Wavelet Coefficients in Human Object Vision: An MEG Study

**DOI:** 10.3389/fncir.2019.00020

**Published:** 2019-04-02

**Authors:** Elaheh Hatamimajoumerd, Alireza Talebpour

**Affiliations:** Department of Computer Science and Engineering, Shahid Beheshti University, Tehran, Iran

**Keywords:** wavelet, MEG data, object recognition, oblique effect, multivariate pattern analysis, orientation

## Abstract

Wavelet transform has been widely used in image and signal processing applications such as denoising and compression. In this study, we explore the relation of the wavelet representation of stimuli with MEG signals acquired from a human object recognition experiment. To investigate the signature of wavelet descriptors in the visual system, we apply five levels of multi-resolution wavelet decomposition to the stimuli presented to participants during MEG recording and extract the approximation and detail sub-bands (horizontal, vertical, diagonal) coefficients in each level of decomposition. Apart from, employing multivariate pattern analysis (MVPA), a linear support vector classifier (SVM) is trained and tested over the time on MEG pattern vectors to decode neural information. Then, we calculate the representational dissimilarity matrix (RDM) on each time point of the MEG data and also on wavelet descriptors using classifier accuracy and one minus Pearson correlation coefficient, respectively. Given the time-courses calculated from performing the Pearson correlation between the wavelet descriptors RDMs and MEG decoding accuracy in each time point, our result shows that the peak latency of the wavelet approximation time courses occurs later for higher level coefficients. Furthermore, studying the neural trace of detail sub-bands indicates that the overall number of statistically significant time points for the horizontal and vertical detail coefficients is noticeably higher than diagonal detail coefficients, confirming the evidence of the oblique effect that the horizontal and vertical lines are more decodable in the human brain.

## Introduction

Feature engineering and mapping input data to a discriminative feature space is the most important and critical part of classical object recognition systems. Various machine learning applications such as texture analysis, image compression and denoising utilize visual features and wavelet representations of images as promising features for object recognition purposes (Strickland and Hahn, 1997; Tieng and Boles, 1997; Khalil and Bayoumi, 2002; Vidal-Naquet and Ullman, 2003; Samani and Moghaddam, 2017; Samani et al., 2018). Performing a two-dimensional wavelet transform on an image provides one approximation and three detail (horizontal, vertical and diagonal) sub-bands representations of images. extracted the shape of pedestrians using only a combination of wavelet coefficients as input features to support vector machine (SVM) classifier. They then could detect the pedestrians in images with different indoor and outdoor backgrounds with a reasonable performance. proposed a Gabor wavelet model as a representation of images which yielded to better object recognition in comparison to applying conventional Gabor filters. Furthermore, wavelet transform has been implemented on diverse types of images from medical to real-world images which demonstrates the capability of this transformation in dealing with computer vision challenges.

On the other hand, among numerous research topics done in neuroscience and psychology regarding the visual processing (Attneave, 1954; Liu et al., 2002; Chaumon et al., 2009; Mamashli et al., 2010), many behavioral and neuroimaging studies on visual perception of different species including humans confirmed in comparison with oblique orientations, cardinal (horizontal or vertical) details of visual stimuli are better resolved in the brain which is known as the oblique effect (Taylor, 1963; Appelle, 1972; Freeman and Thibos, 1975; Poggio and Fischer, 1977; Orban and Vandenbussche, 1979; Essock, 1980; Bonds, 1982; Payne and Berman, 1983; Moskowitz and Sokol, 1985; Heeley et al., 1997; Pantazis et al., 2017). Neural data from different sources of functional neuroimaging modalities such as EEG, MEG and fMRI data have been used to represent this effect in the human visual processing. Furmanski and Engel (2000) used stimuli with cardinal and diagonal orientations in an fMRI study. They found that cardinal orientations generate an increased fMRI response amplitude in V1 area. presented standard and deviated stimuli containing task-irrelevant Gabor patches in an oddball sequence during a tracking task to investigate non-attended orientation anisotropies using ERP (event-related-potential). By recording visual mismatch negativity, they found that there is a difference between the amplitude of ERP evoked by standard and deviated stimuli around 170 ms in occipitotemporal areas as evidence to the existence of the oblique effect. applied multivariate pattern analysis on the Gamma band of MEG data acquired from an experiment using six different grating stimuli. Their results show cardinal orientations are better decoded in the human brain than the oblique ones.

As we described earlier, many studies have been conducted in both the human and machine vision domains to support the idea that the orientation detail is a key factor for object recognition purposes. Here, we built a bridge between a straight machine learning and a pure neuroimaging method in an object recognition challenge. In this study, we used the same stimuli applied in the human object recognition experiment. We extracted wavelet detail coefficients in horizontal, vertical and diagonal orientations to investigate the signatures of different orientations in the human visual system. Furthermore, instead of using basic grating stimuli used in different neuroimaging studies, we employed stimuli containing real-world objects and extracted orientation-related features from them. Our result shows that the oblique effect is evident even with these stimuli. Apart from that, we also studied the temporal neural signature of wavelet approximation coefficients at different levels of decomposition. Due to the downsampling, the approximation coefficients corresponding to higher levels of decomposition contain a denser representation of objects. Therefore, their corresponding timecourses which represent the temporal neural traces of higher level wavelet approximation coefficients peak later.

## Materials and Methods

### Experimental Design, Stimuli, and MEG Data acquisition

We applied all the analysis and inferences on the data of an experiment designed and conducted at MIT by Cichy et al. (2014). During this experiment, 92 stimuli from six distinct categories (human and non-human bodies and faces, natural and artificial images) presented to 16 healthy human participants (*N* = 16) while MEG data was acquired. These images were displayed for 500 ms, with 1.5–2s inter-stimulus-intervals. The participants finished 10–15 MEG runs and every stimulus was shown twice in each run. To read more details see Cichy et al. (2014).

### MEG Signal Preprocessing

MEG data were acquired from 306 sensor channels (Neuromag, Triux, Elekta, Stockholm) (Cichy et al., 2014). To compensate for the head movement, we preprocessed the raw MEG data with Max filter software (Elekta, Stockholm). Then, the resulting signal was denoised and analyzed using the brainstorm software (Tadel et al., 2011). We extracted each trial from 150 ms pre-stimulus onset to 1,000 ms post-stimulus onset (−150, 1,000). Then, we removed the baseline mean for each trial. We also discarded the trials having a peak-to-peak >6,000 fT and detected them as bad trials. Furthermore, a low-pass filter with a cutting frequency of 30 Hz has been used to smoothen the remaining trials. Finally, we utilized the frontal sensors of MEG data to automatically detect the Eyeblink artifacts and remove them by principal component analysis.

### Multivariate Pattern Analysis (MVPA)

**Figure 1:**
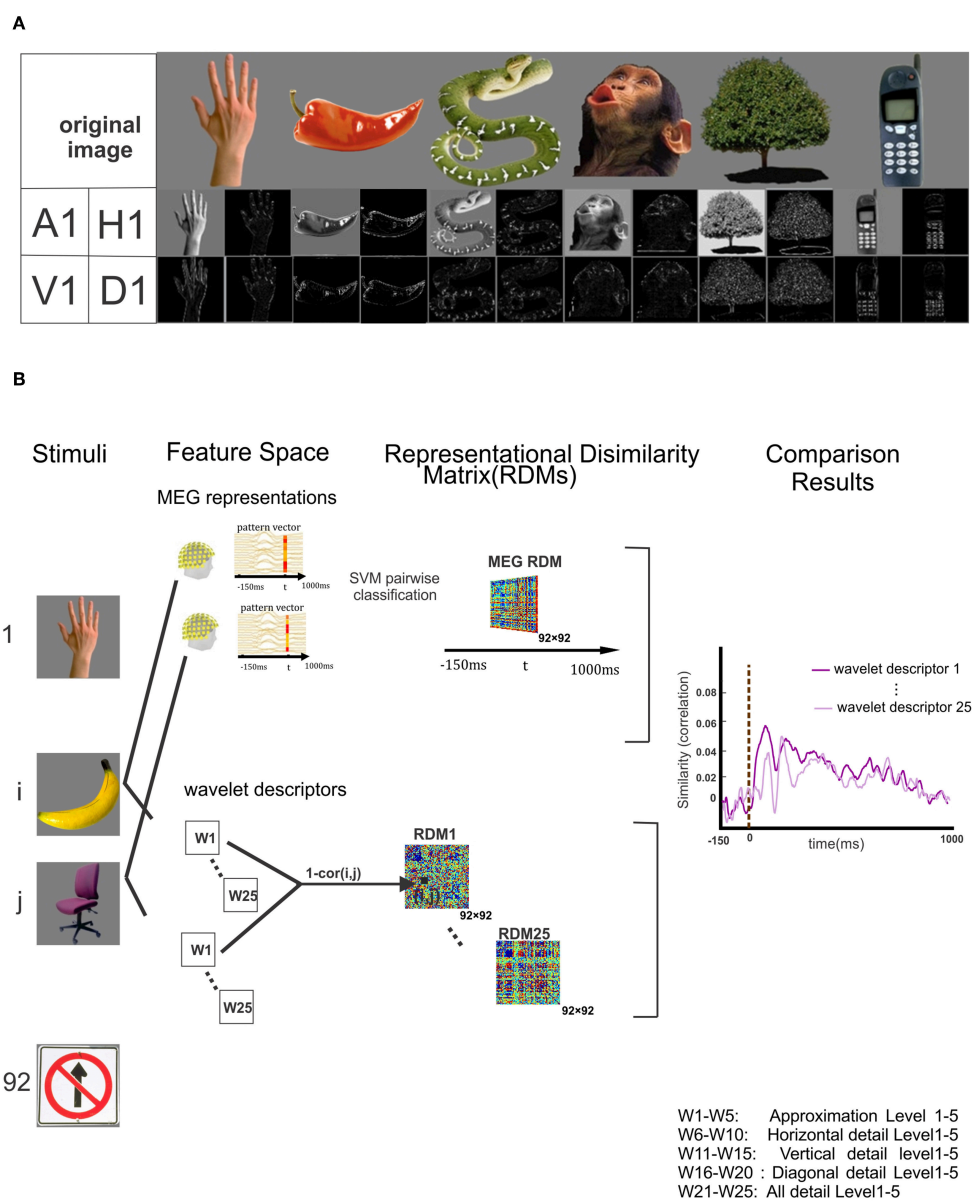
Wavelet transformation on stimuli and block diagram of proposed method. **(A)** Some of the 92 original stimuli and their corresponding wavelet approximation and details coefficients descriptors are displayed in the first row and second row. **(B)** Block diagram of the proposed method. The stimuli are represented in the first column. The upper part of panel shows the schematic of MEG multivariate pattern analysis in which the linear SVM classifier is trained to discriminate between each pair of stimuli at each time point and pairwise decoding accuracy is stored in 92 × 92 representational dissimilarity matrix (RDM). The lower part of the panel shows the wavelet descriptors consisting of Approximation and details (vertical, horizontal and diagonal) descriptors of each stimulus up to five level. For each descriptor, the Spearman correlation between the wavelet descriptors corresponding to every pair of stimuli is calculated, and the results are stored and identified as a wavelet RDM descriptors. All 25 wavelet descriptors are specified in the bottom right corner of this figure. The last column of panel illustrates the time courses resulted from the Spearman correlation MEG RDM in each time point and the RDM of each wavelet descriptors separately.

Multivariate pattern classification is a well-suited approach to decode brain activities associated with different perceptual stimuli. According to this method, if a classifier discriminates between the MEG data of two different stimuli, these two stimuli are separable in the human brain (Wardle et al., 2016; Cichy and Pantazis, 2017; Diedrichsen and Kriegeskorte, 2017; Grootswagers et al., 2017). To measure the perceptual differences of the stimuli, we trained a linear pairwise SVM classifier (Chang and Lin, 2011) at each time point (every millisecond) of the MEG trials associated with every pair of stimuli. In other words, we build the binary linear SVM model using 306-dimensional MEG pattern vectors which are the signal values of all MEG channels. In order to reduce noise and computational load, we permuted the trials randomly and divided them into *K* = 4 groups of 10 trials and averaged the trials within groups, resulting in 4 sub-averaged trials per stimulus. We used K-1 trials per condition for training and held the remaining one which was not used in the training phase, for testing the SVM classifier. This procedure was repeated 100 times to find the SVM classifier performance. The accuracy of pairwise linear SVM classifier is used as a measure of dissimilarity between every pair of the stimuli to populate a 92 × 92 representational dissimilarity matrix (RDM) (Figure 1B, the upper part of the panel). Considering 92 × 92 possible pairs of stimuli and 1,151 time points in each trial, MVPA yields 1,151 symmetric diagonal-undefined MEG RDMs. Having calculated the grand average of every matrix at each time point, we plotted and traced the time-course of object decoding in the human brain.

### Wavelet Feature Descriptors

**Figure 2:**
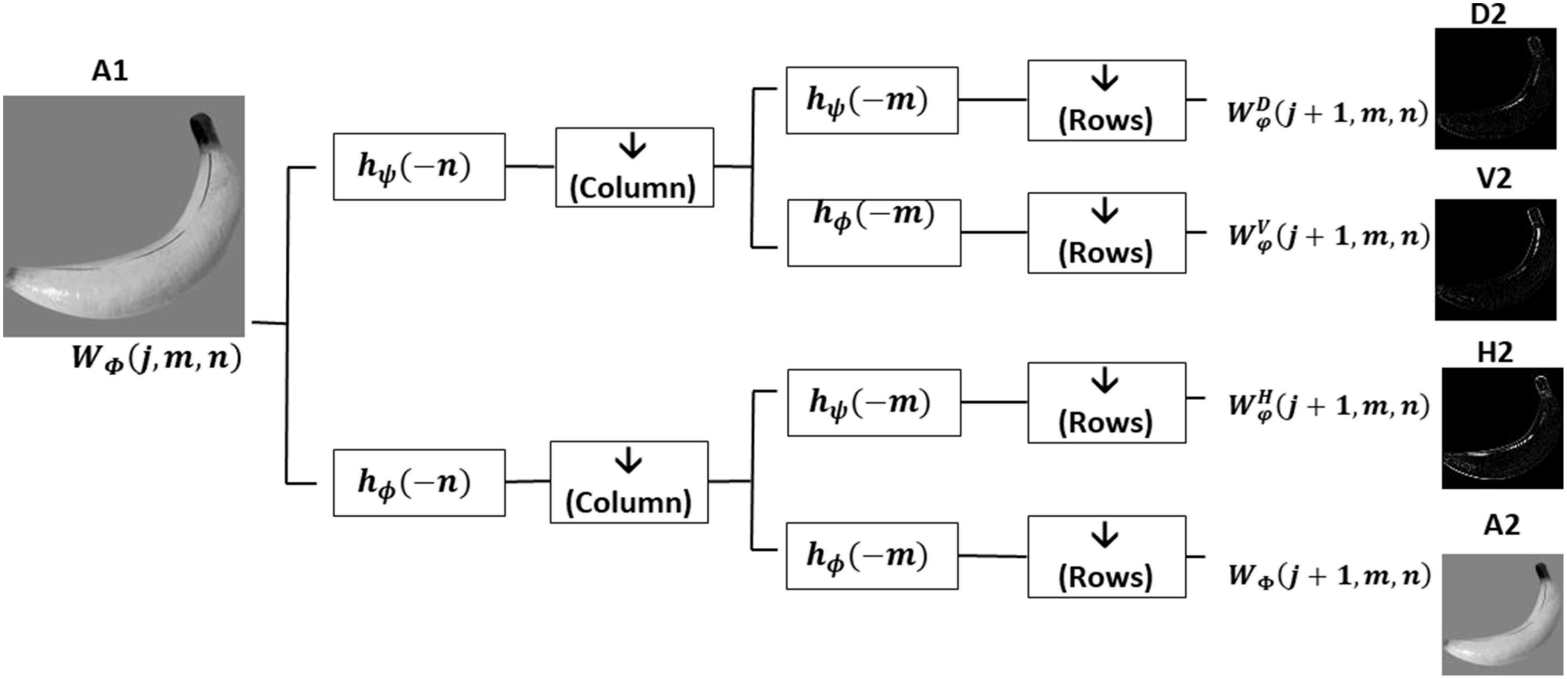
One level of two-dimensional wavelet decomposition on an input image. A sequence of low-pass (*h*_ϕ_) and high-pass (_*h*ψ_) filters, selected from the collection of wavelet basis functions Φ and ψ, are applied on the columns (n) and rows (m) of images. While *h*_ϕ_(*n*) and _*h*ψ_(*n*) shows that the filters are applied in columns, *h*_ϕ_(*n*) and _*h*ψ_(*n*) show that the filters are applied in rows. All filters are followed by a downsampling () by a factor 2 on filtered rows or columns. Applying two low-pass filters (*h*_ϕ_(*n*) and *h*_ϕ_(*m*)) followed by downsampling the sub-image at first level (A1) provides the approximation sub-image of the second level (A2). The second level horizontal and vertical sub images (H2, V2) are calculated by a combination of a low and high-pass filters and downsampling A1 with the specified orders. For extracting the diagonal details descriptors of second level (D2), the high pass filtering and downsampling by factor 2 is sequentially applied to both columns and rows of the sub-image A1.

The multilevel wavelet transform decomposes a complex signal or an image into multiple simpler components which can be studied separately (Ravichandran et al., 2016). Discrete two-dimensional wavelet transform uses a set of discrete scale and translation functions to decompose an image into a set of mutually orthogonal wavelet descriptors (Mallat, 1989; Antonini et al., 1992; Graps, 1995; Stanković and Falkowski, 2003). Equations (1–4) (Ravichandran et al., 2016) define the wavelet transform for calculating the approximation, horizontal, vertical and diagonal sub-bands descriptors respectively. Φ_*j, m, n*_(*x, y*) and $\varphi_{j,m,n\;}^{i},$ defined in Equations (5, 6), describe the two-dimensional wavelet functions of scale (level) *j* at pixel in row *m* and column *n* of an input image with *M* rows and *N* columns of pixels. These functions act as low-pass and high-pass filters followed by downsampling. Superscript *i* in the Equation (6) shows the orientation of wavelet details coefficients which can be horizontal (H), vertical (V), and diagonal (D). The block diagram in Figure 2 describes one level of two-dimensional wavelet decomposition on an input image. According to this diagram, two finite impulse response (FIR) low-pass (*h*_ϕ_) and high-pass (*h*_ψ_) filters, selected from the collection of wavelet basis functions Φ and ψ defined in Equations (5, 6), are applied to columns or rows of an input image. To get the wavelet approximation coefficients, a sequence of two low-pass filters followed by downsampling (by a factor of 2) is applied to columns and rows of the image. Horizontal and vertical detail sub-images are calculated by a combination of a low and high-pass filter and downsamplings. As Figure 2 shows, swapping the order of low and high-pass filters switches the resulting orientation of detail descriptors. For extracting the detail descriptors, a sequence of identical high pass filtering and downsampling is applied to both columns and rows of an image. To compute the wavelet descriptors of level *j*+*1*, approximation wavelet descriptor of level j is used as an input image and the whole process is repeated in the same mentioned manner.

(1) $$W_{\Phi }\left(j,m,n\right)=\frac{1}{\sqrt{MN}}\sum_{x=0}^{M-1}\sum_{y=0}^{N-1}I(x,y){\text{Φ}}_{j_{o,m,n\;}}$$

(2) $$W_{\varphi }^{H}\left(j,m,n\right)=\frac{1}{\sqrt{MN}}\sum_{x=0}^{M-1}\sum_{y=0}^{N-1}I\left(x,y\right)\varphi_{j,m,n}^{H}$$

(3) $$W_{\varphi }^{V}\left(j,m,n\right)=\frac{1}{\sqrt{MN}}\sum_{x=0}^{M-1}\sum_{y=0}^{N-1}I\left(x,y\right)\varphi_{j,m,n}^{V}$$

(4) $$W_{\varphi }^{D}\left(j,m,n\right)=\frac{1}{\sqrt{MN}}\sum_{x=0}^{M-1}\sum_{y=0}^{N-1}I\left(x,y\right)\varphi_{j,m,n}^{D}$$

(5) $${\text{Φ}}_{j,m,n}\left(x,y\right)=2^{\frac{j}{2}}\text{Φ}\left(2^{j}x-m,2^{\frac{j}{2}}y-n\right)$$

(6) $$\varphi_{j,m,n}^{i}\left(x,y\right)=2^{\frac{j}{2}}\varphi \left(2^{j}x-m,2^{\frac{j}{2}}y-n\right)\;i=H,V,\;D$$

(7) $$\text{Φ}\left(t\right)=\{\;\begin{array}{c} 1\;0\leq t<\frac{1}{2}\;\\ -1\;\frac{1}{2}\leq t<\;1\;\\ 0\;otherwise \end{array}$$

(8) $$\varphi \left(t\right)=\{\begin{array}{c} 1\;0\leq t<1\\ 0\;otherwise \end{array}$$

We employed two-dimensional Haar wavelet on the gray-scaled stimuli to decompose them into four orthogonal sub-bands. The mother Haar wavelets Φ and φ are defined in Equations (7, 8). The first row of Figure 1 depicts some examples of the original images and the second one illustrates their corresponding level-one approximation (A1) and horizontal(H1), vertical(V1) and diagonal(D1) high-frequency details. According to the Block Diagram in Figure 1, each level of decomposition provides four wavelet components whose rows and columns resolutions are half of the previous level. All the original images have 175 rows and columns. Due to downsampling of the images in both rows and columns at each level of decomposition, the size of approximation and detail sub-images in the fifth level is 6 × 6. All the approximation and detail sub-bands matrices calculated in different levels are flattened and rearranged into vectors separately. To study the overall neural information decoded in the details in each level, we concatenated the horizontal, vertical and diagonal details vectors corresponding to each stimulus as an additional descriptor and called it 'All details' descriptor. In general, after 5 levels of decomposition with 5 different wavelet descriptors per level, a total number of 25 wavelet descriptor vectors are determined for each stimulus. As shown in the bottom right corner of Figure 1, W1 to W25 represent the wavelet descriptors for different sub-band at different levels.

### Wavelet Coefficients RDM

Representational dissimilarity matrix (RDM) maps the descriptors into a common space and provides the overall representational information of each wavelet descriptors. For each wavelet descriptor, we computed 1 min Pearson's rho as a dissimilarity measure between each pair of stimuli wavelet descriptors to construct a 92 × 92 RDM matrix. Since we have 25 different wavelet descriptors per stimulus, this process results in 25 RDM matrices corresponding to 25 wavelet descriptors (Figure 1B lower part of the panel).

### Representational Similarity Analysis

To trace the neural signature of stimulus wavelet descriptors in the visual system, we used the representational similarity analysis (RSA). We mapped MEG data of each time point to the representational space (92 × 92 RDMs) using the MVPA. Similarly, we also mapped the 25 wavelet descriptors to the representational space of 92 × 92 RDMs. Therefore, in this common two-dimensional (92 × 92) matrix space, we can compare the wavelet RDMs with the neural RDMs using the two-dimensional Spearman correlation between wavelet descriptors RDMs and the MEG RDMs in each time point. This results in 25 time courses representing the temporal neural traces of the wavelet descriptors in the human object recognition (Figure 1B).

### Statistical Testing

In order to estimate the significant time points of the time series, we performed non-parametric signed permutation statistical test (Pantazis et al., 2005; Nichols, 2012) broadly used in neuroimaging studies (Hayasaka and Nichols, 2004; Mirman et al., 2016; Mohsenzadeh et al., 2018). Permutation and bootstrap were done with a sample size (*n* = 16) equal to the number of subjects. We used 1,000 bootstrap samples. In each bootstrap sample, we chose 16 time series with replacement among all subject time courses and we estimated the significant time points and onset time. To estimate the significant time points and assess the statistical significance of the time series, we performed the sign permutation test. Since the time series carry the results of correlation between MEG pattern classification and Wavelet descriptors, the null hypothesis indicates no signal or dynamics in the time series. To do the sign permutation test, we randomly permuted the labels of MEG data (conditions labels). Therefore, the subjects' responses were randomly multiplied by +1 or −1. The operations (permutation and bootstrapping with sample size 16) were repeated for 1,000 times which led us to provide a one-dimensional *p*-value statistical map. Then, we performed the cluster correction test to regularize the error across all the time points. The cluster definition threshold was set to 0.05. According to this test, if the size of connected time points (clusters) was greater than the threshold these time points were considered significant.

We used bootstrapping to test and estimate the peak and onset latencies of the time courses. The time series for each subject were bootstrapped and averaged across the subjects 1,000 times. The standard error of measurement (SEM) and 95% confidence intervals are defined based on the distribution of obtained Peaks or onsets of all bootstrap samples.

## Results and Discussion

We explored the temporal relation between the brain activity and wavelet representations of real-world images. The signature results of wavelet components in the human brain during an object vision task are presented separately for five levels of wavelet decomposition. These components consist of approximation and sparse detail sub-bands of two dimensional Haar wavelet transform. We also investigated the neural information encoded in the visual system regarding wavelet descriptors of images.

### Representational Similarity Comparison of Brain Data With Wavelet Approximation and Detail

**Figure 3:**
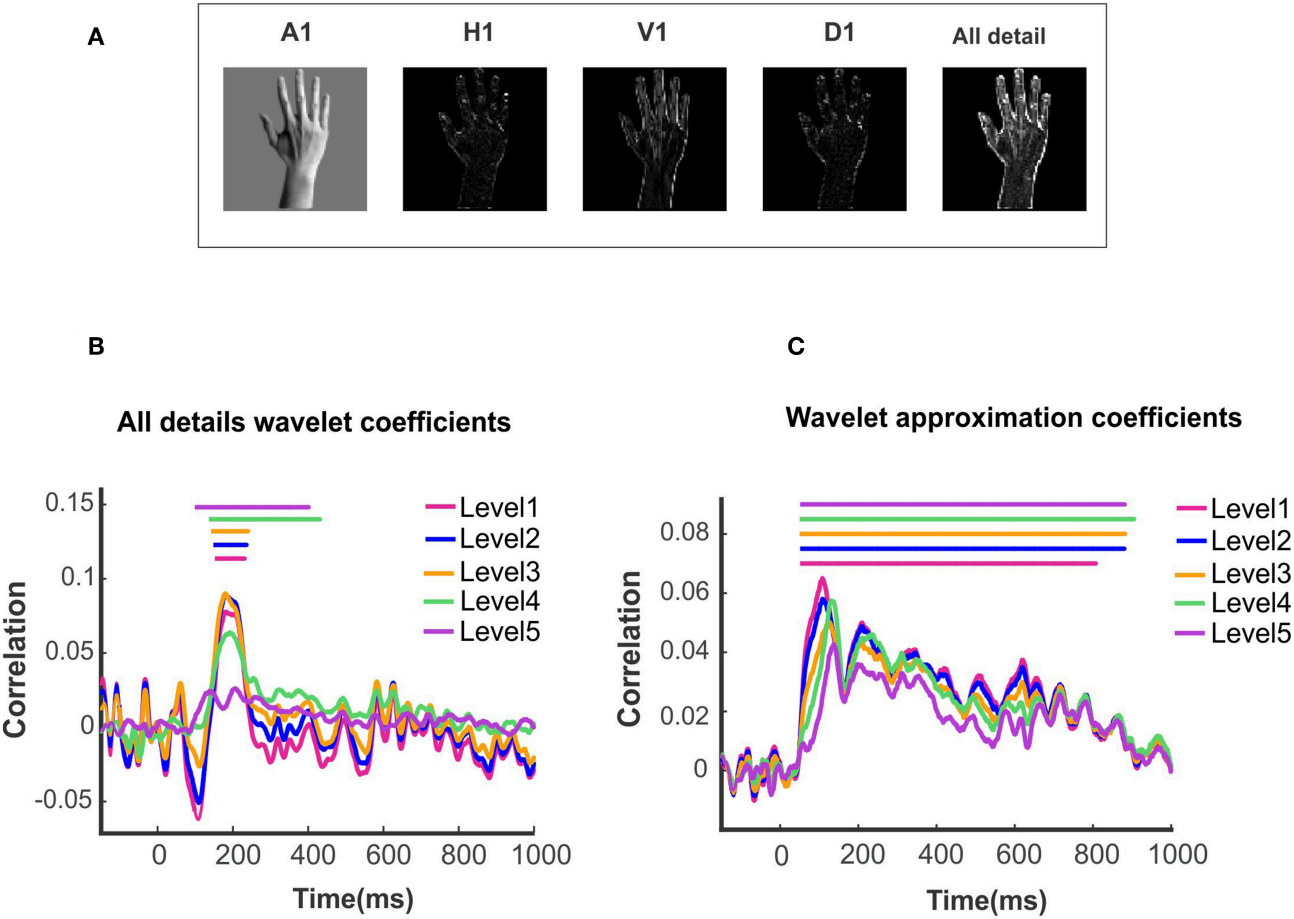
Wavelet approximation and detail information decoded in MEG signal. **(A)** An example of first level wavelet descriptors: From left to right, wavelet approximation, horizontal, vertical, diagonal and all detail descriptors. **(B)**Time courses illustrating the similarity between wavelet details of different levels and brain representation of images. **(C)**Time courses illustrating the similarity between wavelet approximation of different levels and brain representation of images. Solid lines above the time courses demonstrated the significant time points evaluated with two-sided sign permutation test (*N* = 16, cluster definition threshold *P* = 0.05 and cluster definition *P* = 0.05).

First, we investigate how the wavelet representation (approximation and details) of images at different levels of composition are encoded in neural data. With this aim, we use multivariate pattern analysis on MEG data to compute the neural representations and calculate the wavelet descriptors of images and create their corresponding RDM. One example of the approximation (A1) and detail descriptors including horizontal (H1), vertical (V1), diagonal (D1) and ‘all details' are shown from left to right on Figure 3A. Figures 3B,C shows the time courses generated from performing the Spearman correlation between MEG RDM at each time point and wavelet approximation as well as details RDMs. Color-coded solid lines above the time courses mark the significant time points for each curve. All the significant time points are found with non-parametric permutation statistical tests using cluster defining threshold *P* < *0.05*, and corrected significance level *P* < *0.05* (N = 16). As can be seen in Figure 3C, the curves representing the neuro-dynamics of wavelet approximation of all five levels maintain the same trends and time courses. With regard to the significant time points, we found that the neural signatures of wavelet approximation coefficients are sustained, while they are transient for details descriptors. Research on the spatiotemporal dynamics of object recognition in the human brain (Cichy et al., 2014) demonstrates that neuronal processing of objects can be both transient and persistent. As Figure 3A illustrates, all-detail descriptor which aggregates the detail descriptors in three orientations (horizontal, vertical and diagonal) captures the overall shape and some categorical information of objects. For this reason, it can act as a discriminant feature in the human object recognition, but it still lacks other low, mid and high-level features. However, wavelet approximation descriptors contain a broader range of high-level semantics of images which may be processed and maintained in the later visual processing areas of the human brain such as Inferior-temporal (IT), Fusiform, and Parahippocampal cortex (PHC).

### Comparison of Peak Latencies in Different Levels of Wavelet Approximation Time Courses

Given sustained neural information of wavelet approximation time courses at all five levels, we further asked how categorical information is represented in different levels. To understand that, we estimated the peak latency of these time courses using the signed permutation test (*N* = 16; *p* < 0.05). We found that, as the level of wavelet approximation coefficients increases, peak latencies of its corresponding time course occurs later. This can be explained by the fact that the sub-band images extracted from higher levels of wavelet decomposition contain less sparsity and signify a denser representation of the stimuli which accentuate semantic information decoded in the stimuli.

**Figure 4:**
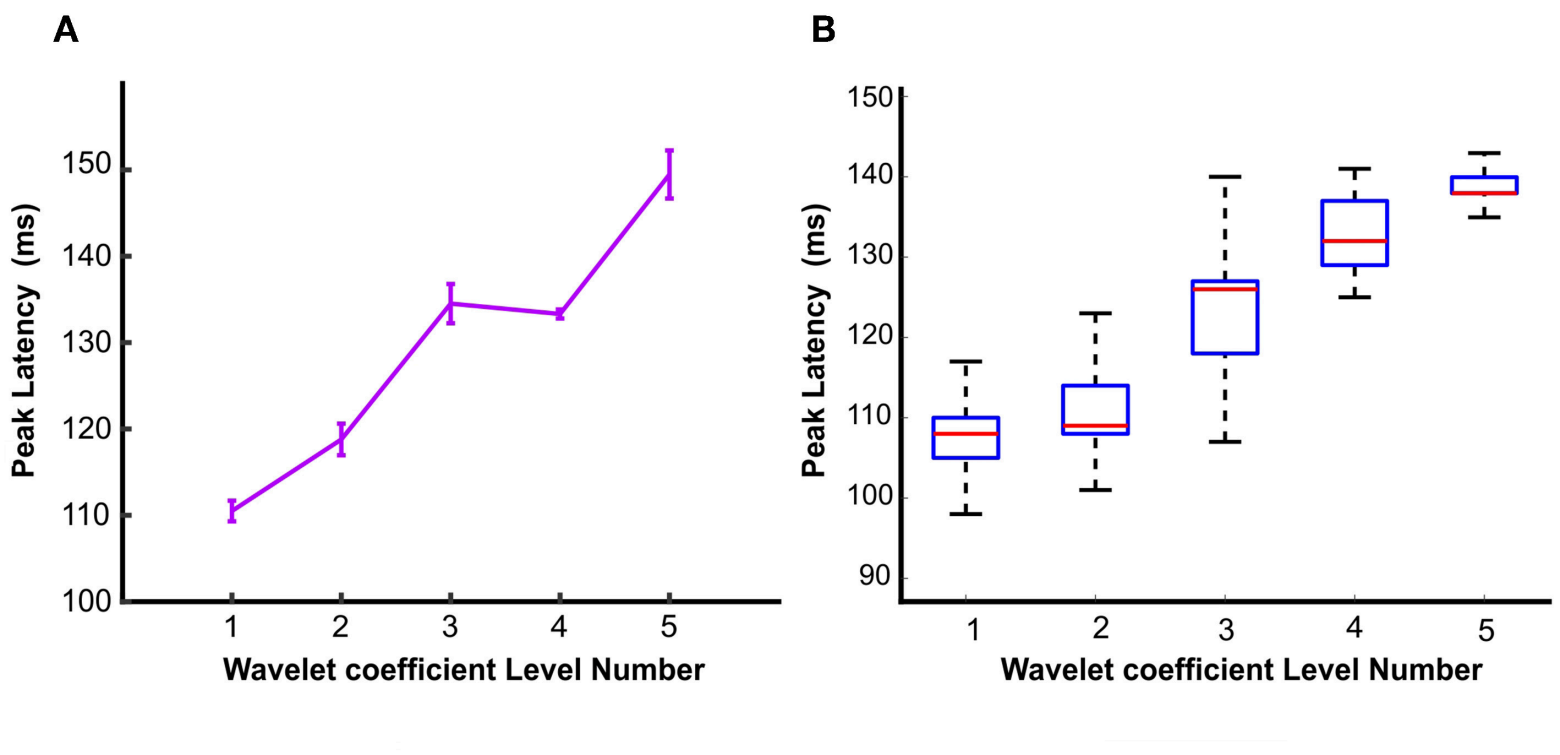
Peak latencies of wavelet approximation time course at different levels represented by **(A)** Error bar and **(B)** Box plot. Peak latencies increased with the level number evaluated by signed permutation test non-parametric permutation statistical tests using cluster defining threshold *P* < 0.05, and corrected significance level *P* < 0.05 (*N* = 16). Error bar represents the standard error of the mean calculated by 1,000 times bootstrapping the participant sample. To remove the outliers, the top and bottom 5% of data points have been discarded.

**Figure 5:**
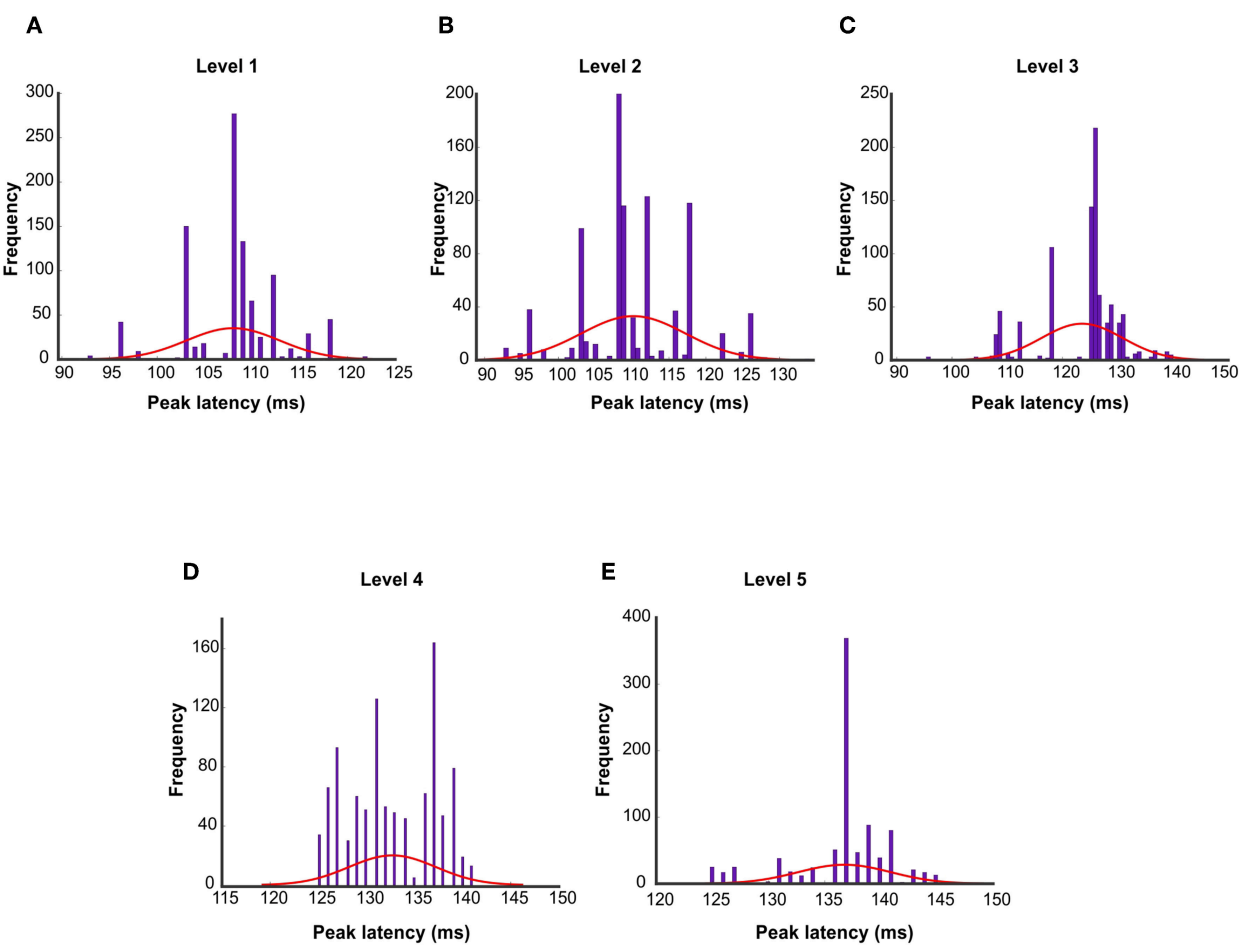
Histogram of peak latencies of wavelet approximation time course at levels Level 1 to Level 5 **(A–E)** estimated by 1,000 times bootstrapping the participant sample. The red curves show the normal density functions fitted on the histograms. The mean peak latencies of the distributions are 108, 110,123, 132, 136 ms for level 1–level 5 respectively which demonstrates the peak latencies increased with the decomposition level number.

The first and second columns of Table 1 report the peak latency of wavelet approximation time courses with 95% confidence interval and (mean ± SEM) in which SEM represents the standard error of measurement. The third column reports the onset time. Figures 4A,B illustrates the peak latencies of wavelet approximation coefficient at different levels of decomposition with error bar and box plot using bootstrap test. To remove the outliers, the top and bottom 5% of data points have been discarded. Figure 5 displays the histograms of peak latencies at different levels using 1,000 bootstrap samples and the red curves show the normal density functions fitted on the histograms. Since the object recognition and categorization in the human brain occurs earlier than 150 ms after the stimulus onset Thorpe et al. (1996) Isik et al. (2013), we kept the first group of data which shows the histogram of peak latency happening earlier or equal to 150 ms and we discarded the data points with peak latency >150 ms which contain a small portion of all the data points. The mean peak latencies of the distributions fitted on histograms in Figure 5 are 108, 110,123, 132, 136 ms for level 1 to level 5, respectively. Similarly, as Figures 4A,B confirms, the peak latencies of wavelet approximation time-courses increase with level. This suggests that approximation descriptors at higher levels carry more categorical and semantic visual information required to be processed in later visual processing areas across the ventral stream pathway.

**Table 1:** Peak latency and onset of wavelet descriptors time courses of approximation coefficients for 5 levels.

**Approximation coefficients**	**Peak latency(conf95)**	**Mean peak ± SEM**	**Onset(conf95)**
Level1	108 (95–208)	110.5 ± 1.2	50(43–54)
Level2	108 (96–210)	118.7 ± 1.8	50(41–53)
Level3	126 (108–213)	134.5 ± 2.3	49(41–54)
Level4	131(125–140)	133.3 ± 0.5	49(41–68)
Level5	137(125–254)	149.4 ± 2.8	50(45–95)

### Representational Similarity Analysis Infers the Oblique Effect for Wavelet Details Descriptors

**Figure 6:**
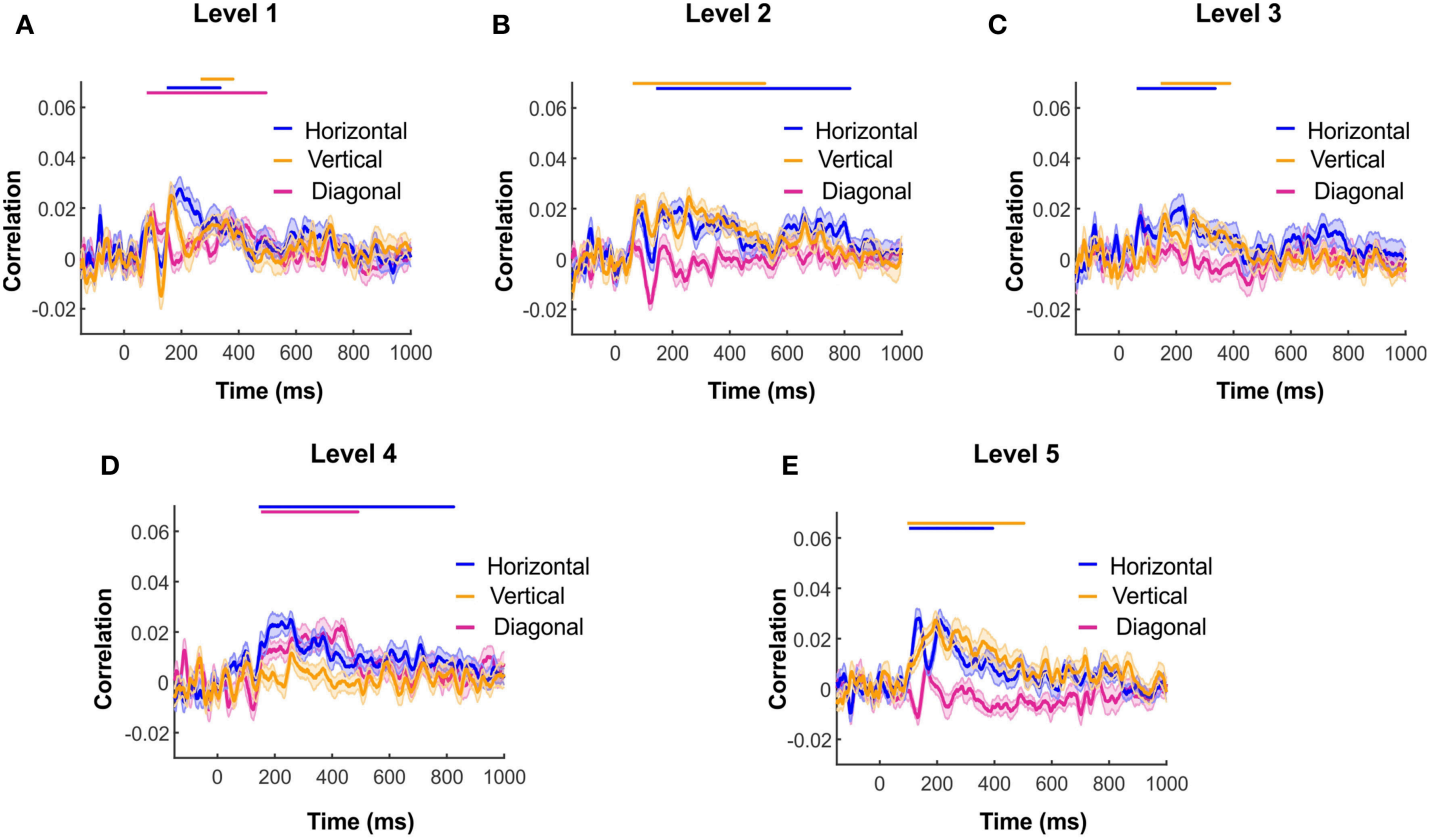
Wavelet details information in different orientations decoded in MEG time course of Spearman correlation between MEG RDM with horizontal, vertical and diagonal wavelet details descriptors RDM of Level 1 to 5 **(A–E)** respectively. Shaded color-coded areas around the curves in Figure 6 **(A–E)** shows the standard deviation of time courses over 1,000 bootstrap sampling with sample size *N* = 16.

**Figure 7:**
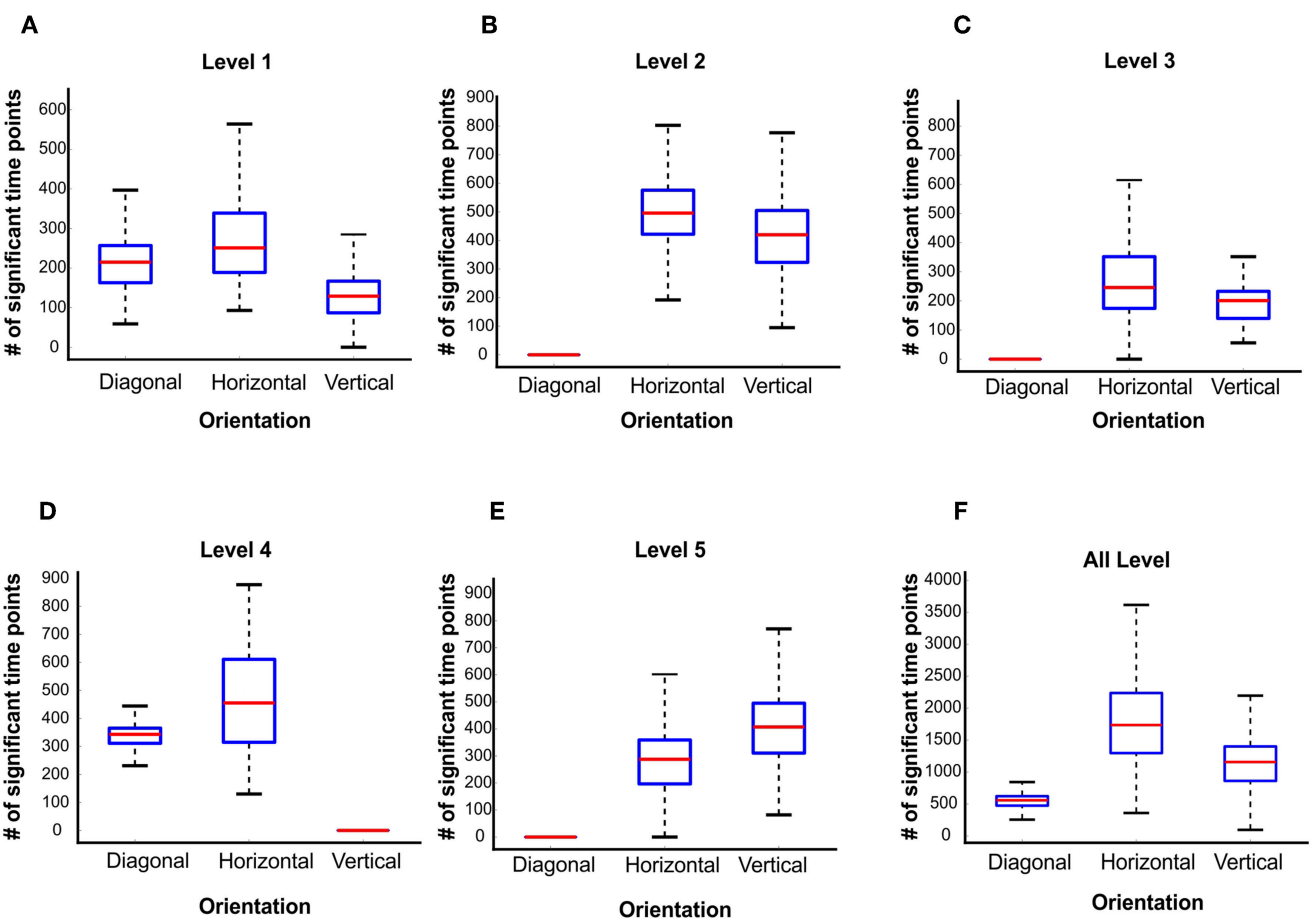
Number of significant time points of the time courses of wavelet details descriptors at level 1 to level 5 **(A–E)**. A number of 1,000 bootstrapping is performed on the participant sample and the significant time points evaluated with two-sided sign permutation test (*N* = 16, cluster definition threshold *P* = 0.05 and cluster definition *P* = 0.05). **(F)** The summation of all significant time points for different orientations.

To further study the signature of wavelet details coefficients of different orientations, we calculated the wavelet details RDM for each orientation separately. Figures 6A–E illustrate the time courses of Spearman correlation between MEG RDMs and horizontal, vertical and diagonal wavelet detail RDMs for Levels 1–5. In each time point, we performed 1,000 bootstrap samples (sample size *N* = 16) with replacement among all subject time courses and averaged them across the subjects. Shaded color-coded areas around the curves in Figure 6A–E shows the standard deviation of the time courses over 1,000 bootstrap samples. Similar to Figure 3, color-coded solid lines above the time courses express the significant time points. The box plots in Figures 7A–E illustrate the number of significant time points of time courses corresponding to diagonal, horizontal and vertical detail descriptors using bootstrap sampling. Figure 7F represents the summation of all significant time point for different orientations. As shown, the overall number of significant time points of the horizontal and vertical wavelet time courses is noticeably greater than the diagonal wavelet time course. This suggests that vertical and horizontal details are represented stronger in the human brain.

The majority of experimental researchers have designed or used the specific stimuli such as grating stimuli to study the oblique effect. employed MVPA to investigate the decoding of various orientations with a set of six different grating stimuli. Their results confirmed the oblique effect in the human visual system. In our study, we inferred the oblique effect on the human visual system by estimating the wavelet details descriptors of the real-world images as representatives of orientations. This inference is based on the number of significant time points which can be interpreted as the time points in which there is a significant and meaningful correlation of MEG data and its corresponding wavelet orientation details.

## Conclusion

We explored the neuro-dynamic of wavelet approximation and details sub-bands in human vision. Although orientation-specific stimuli were not used in this study, our results revealed that MVPA is a well-suited approach for inference of the implicit oblique effect in the human visual system. Furthermore, we found that while the signature of wavelet details descriptors was transient, there was a sustained significant correlation between the approximation descriptors and neural data. The result of our study on the time course of wavelet approximation coefficient indicated that the peak latencies of correlation time series increased with the approximation level. This effect implies that decreasing the size of images and increasing the level of wavelet approximation coefficients causes a reduction of sparsity and highlights semantic and categorical information of objects in the human visual system.

## Ethics Statement

The stimuli and MEG data of this study are provided by Cichy et al. (2014). Their study was in compliance with the Declaration of Helsinki and approved by the Institutional Review Board of the Massachusetts Institute of Technology.

## Author Contributions

EH analyzed the MEG data and stimuli and wrote the manuscript. AT assisted with the interpretation of results and advised on analysis and writing process.

### Conflict of Interest Statement

The authors declare that the research was conducted in the absence of any commercial or financial relationships that could be construed as a potential conflict of interest.

## References

[B1] Antonini M. Barlaud M. Mathieu P. Daubechies I. (1992). Image coding using wavelet transform. IEEE Trans. Image Proc. 1, 205–220. 10.1109/83.136597 18296155

[B2] Appelle S. (1972). Perception and discrimination as a function of stimulus orientation: the” oblique effect“ in man and animals. Psychol. Bull. 78, 266–278. 10.1037/h0033117 4562947

[B3] Attneave F. (1954). Some informational aspects of visual perception. Psychol. Rev. 61, 183–193. 10.1037/h0054663 13167245

[B4] Bonds A. (1982). An “oblique effect” in the visual evoked potential of the cat. Exp. Brain Res. 46, 151–154. 7067788 10.1007/BF00238110

[B5] Chang C.-C. Lin C.-J. (2011). LIBSVM: a library for support vector machines. ACM Trans. Intel. Syst. Technol. 2, 27 10.1145/1961189.1961199

[B6] Chaumon M. Schwartz D. Tallon-Baudry C. (2009). Unconscious learning versus visual perception: dissociable roles for gamma oscillations revealed in MEG. J. Cogn. Neurosci. 21, 2287–2299. 10.1162/jocn.2008.21155 18855554

[B7] Cichy R. M. Pantazis D. (2017). Multivariate pattern analysis of MEG and EEG: a comparison of representational structure in time and space. Neuroimage 158, 441–454. 10.1016/j.neuroimage.2017.07.023 28716718

[B8] Cichy R. M. Pantazis D. Oliva A. (2014). Resolving human object recognition in space and time. Nat. Neurosci. 17, 455–462. 10.1038/nn.3635 24464044 PMC4261693

[B10] Diedrichsen J. Kriegeskorte N. (2017). Representational models: a common framework for understanding encoding, pattern-component, and representational-similarity analysis. PLoS Comput. Biol. 13:e1005508. 10.1371/journal.pcbi.1005508 28437426 PMC5421820

[B11] Essock E. A. (1980). The oblique effect of stimulus identification considered with respect to two classes of oblique effects. Perception 9, 37–46. 10.1068/p090037 7360612

[B12] Freeman R. D. Thibos L. N. (1975). Visual evoked responses in humans with abnormal visual experience. J. Physiol. (Lond). 247, 711–724. 10.1113/jphysiol.1975.sp010953 1142304 PMC1309494

[B13] Furmanski C. S. Engel S. A. (2000). An oblique effect in human primary visual cortex. Nat. Neurosci. 3, 535–536. 10.1038/75702 10816307

[B14] Graps A. (1995). An introduction to wavelets. IEEE Comput. Sci. Eng. 2, 50–61. 10.1109/99.388960

[B15] Grootswagers T. Wardle S. G. Carlson T. A. (2017). Decoding dynamic brain patterns from evoked responses: a tutorial on multivariate pattern analysis applied to time series neuroimaging data. J. Cogn. Neurosci. 29, 677–697. 10.1162/jocn_a_01068 27779910

[B16] Hayasaka S. Nichols T. E. (2004). Combining voxel intensity and cluster extent with permutation test framework. Neuroimage 23, 54–63. 10.1016/j.neuroimage.2004.04.035 15325352

[B17] Heeley D. W. Buchanan-Smith H. M. Cromwell J. A. Wright J. S. (1997). The oblique effect in orientation acuity. Vision Res. 37, 235–242. 10.1016/S0042-6989(96)00097-1 9068823

[B18] Isik L. Meyers E. M. Leibo J. Z. Poggio T. (2013). The dynamics of invariant object recognition in the human visual system. J. Neurophysiol. 111, 91–102. 10.1152/jn.00394.2013 24089402 PMC4280161

[B19] Khalil M. I. Bayoumi M. M. (2002). Affine invariants for object recognition using the wavelet transform. Pattern Recognit. Lett. 23, 57–72. 10.1016/S0167-8655(01)00102-7

[B20] Liu J. Harris A. Kanwisher N. (2002). Stages of processing in face perception: an MEG study. Nat. Neurosci. 5, 910–916. 10.1038/nn909 12195430

[B21] Mallat S. G. (1989). A theory for multiresolution signal decomposition: the wavelet representation. IEEE Trans. Pattern Anal. Mach. Intell. 11, 674–693. 10.1109/34.192463

[B22] Mamashli F. Ahmadlu M. Golpayegani M. R. Gharibzadeh S. (2010). Detection of attention using chaotic global features. J. Neuropsych. Clin Neurosci. 22, 247. e220. 10.1176/appi.neuropsych.22.2.247-m.e20 20463133

[B23] Mirman D. Landrigan J. F. Kokolis S. Verillo S. Ferrara C. (2016). Permutation-based cluster size correction for voxel-based lesion-symptom mapping. arXiv Preprint arXiv:1606.00475. 10.1016/j.neuropsychologia.2017.08.025 PMC5826816 28847712

[B24] Mohsenzadeh Y. Qin S. Cichy R. Pantazis D. (2018). Ultra-Rapid serial visual presentation reveals dynamics of feedforward and feedback processes in the ventral visual pathway. eLife 21:e36329 10.7554/eLife.36329 PMC6029845 29927384

[B25] Moskowitz A. Sokol S. (1985). Effect of stimulus orientation on the latency and amplitude of the VEP. Invest. Ophthalmol. Vis. Sci. 26, 246–248. 3972506

[B26] Nichols T. E. (2012). Multiple testing corrections, nonparametric methods, and random field theory. Neuroimage 62, 811–815. 10.1016/j.neuroimage.2012.04.014 22521256

[B27] Orban G. Vandenbussche E. (1979). Behavioural evidence for the oblique effect in the cat [proceedings]. J. Physiol. 295, 15P–16P. 521918

[B28] Pantazis D. Fang M. Qin S. Mohsenzadeh Y. Li Q. Cichy R. M. (2017). Decoding the orientation of contrast edges from MEG evoked and induced responses. NeuroImage 180, 267–279. 10.1016/j.neuroimage.2017.07.022 28712993

[B29] Pantazis D. Nichols T. E. Baillet S. Leahy R. M. (2005). A comparison of random field theory and permutation methods for the statistical analysis of MEG data. Neuroimage 25, 383–394. 10.1016/j.neuroimage.2004.09.040 15784416

[B30] Payne B. R. Berman N. (1983). Functional organization of neurons in cat striate cortex: variations in preferred orientation and orientation selectivity with receptive-field type, ocular dominance, and location in visual-field map. J. Neurophysiol. 49, 1051–1072. 10.1152/jn.1983.49.4.1051 6854357

[B31] Poggio G. F. Fischer B. (1977). Binocular interaction and depth sensitivity in striate and prestriate cortex of behaving rhesus monkey. J. Neurophysiol. 40, 1392–1405. 10.1152/jn.1977.40.6.1392 411898

[B31a] Ravichandran D. Nimmatoori R. Gulam Ahamad M. (2016). Mathematical representations of 1D, 2D and 3D wavelet transform for image coding. Int. J. Adv. Comput. Theory Eng. 5, 1–8.

[B32] Samani Z. R. Guntuku S. C. Moghaddam M. E. Preotiuc-Pietro D. Ungar L. H. (2018). Cross-platform and cross-interaction study of user personality based on images on twitter and flickr. PLoS ONE 13:e0198660. 10.1371/journal.pone.0198660 29995955 PMC6040697

[B33] Samani Z. R. Moghaddam M. E. (2017). A knowledge-based semantic approach for image collection summarization. Multimed. Tools Appl. 76, 11917–11939. 10.1007/s11042-016-3840-1

[B34] Stanković R. S. Falkowski B. J. (2003). The haar wavelet transform: its status and achievements. Comput. Electric. Eng. 29, 25–44. 10.1016/S0045-7906(01)00011-8

[B35] Strickland R. N. Hahn H. I. (1997). Wavelet transform methods for object detection and recovery. IEEE Trans. Image Proc. 6, 724–735. 10.1109/83.568929 18282965

[B36] Tadel F. Baillet S. Mosher J. C. Pantazis D. Leahy R. M. (2011). Brainstorm: a user-friendly application for MEG/EEG analysis. Comput. Intell. Neurosci. 2011, 8:879716 10.1155/2011/879716 PMC3090754 21584256

[B37] Taylor M. M. (1963). Visual discrimination and orientation. JOSA 53, 763–765. 10.1364/JOSA.53.000763 13993632

[B38] Thorpe S. Fize D. Marlot C. (1996). Speed of processing in the human visual system. Nature 381, 520–522. 10.1038/381520a0 8632824

[B39] Tieng Q. M. Boles W. (1997). Recognition of 2D object contours using the wavelet transform zero-crossing representation. IEEE Trans. Pattern Anal. Mach. Intell. 19, 910–916. 10.1109/34.608294

[B40] Vidal-Naquet M. Ullman S. (2003). Object Recognition with Informative Features and Linear Classification in Proceedings Ninth IEEE International Conference on Computer Vision (Nice), 281.

[B41] Wardle S. G. Kriegeskorte N. Grootswagers T. Khaligh-Razavi S. M. Carlson T. A. (2016). Perceptual similarity of visual patterns predicts dynamic neural activation patterns measured with MEG. Neuroimage 132, 59–70. 10.1016/j.neuroimage.2016.02.019 26899210

